# Minimally important difference of the Child Oral Health Impact Profile for children with orofacial anomalies

**DOI:** 10.1186/s12955-016-0544-1

**Published:** 2016-10-03

**Authors:** Ryan Richard Ruff, Lacey Sischo, Hillary L. Broder

**Affiliations:** 1 Department of Epidemiology and Health Promotion, New York University College of Dentistry, 433 First Avenue, Room 712, New York, NY USA; 2 College of Global Public Health, New York University, New York, NY USA; 3 Department of Cariology and Comprehensive Care, New York University College of Dentistry, New York, NY USA

**Keywords:** Oral health-related quality of life, Minimally important difference, Cleft

## Abstract

**Background:**

The Child Oral Health Impact Profile (COHIP) is an instrument designed to measure the self-reported oral health-related quality of life of children between the ages of 8 and 15, including domains for oral health, functional well-being, social-emotional well-being, school environment and self-image. The purpose of this study was to estimate the minimally important difference (MID) of the COHIP for patients with cleft lip/palate.

**Methods:**

Data from a 6-year, prospective, longitudinal cohort study of children with cleft lip/palate were analyzed to estimate the MID. Analysis was restricted to patients with data at baseline and first follow-up and not receiving a surgical intervention in the intervening years (*N* = 281). MIDs were estimated via the anchor-based method, using the Global Assessment of Change, and the effect size distribution method.

**Results:**

Based on the distributional method, the minimally important differences were 0.16 (oral health), 0.12 (functional), 0.22 (social-emotional), 0.21 (school environment) and 0.19 (self-image). MID anchor estimates for COHIP domains ranged from −0.32 to 0.84. The anchor-based and effect size MID estimates for the overall COHIP score were 2.95 and 0.25, respectively.

**Conclusion:**

The minimally important difference of the Child Oral Health Impact Profile is recommended for interpreting clinically meaningful change in patients with cleft lip/palate.

## Background

Children born with cleft lip/palate typically require multiple surgeries and ongoing evaluations that extend well into adolescence and young adulthood [1]. Surgical interventions can include secondary palatal surgeries for improved speech, lip and nose revisions for improved facial performance, and alveolar bone graft surgery for improved functional well-being (e.g., tooth and bone development) [2]. Most children with cleft have multiple surgeries before they complete treatment and/or reach adulthood, yet little is known about the long-term effects of these interventions on patient-reported outcomes such as quality of life. While it is often assumed by surgeons, caregivers, and patients that cleft-related surgeries have a positive impact on patients’ lives, this assumption may be unfounded. Patients may experience treatment burnout, a phenomenon studied in other chronic conditions such as diabetes [3] and general orthodontic treatment [4], or fail to derive substantive benefit from treatment beyond measured clinical outcomes.

Oral health-related quality of life (OHRQoL), as a “multidimensional construct that includes a subjective evaluation of an individual’s oral health, functional well-being, emotional well-being, expectations and satisfaction with care, and sense of self” [5], may be particularly salient to children with orofacial anomalies. While there are multiple OHRQoL measures available for patient assessment in oral health, the Child Oral Health Impact Profile (COHIP) was specifically designed for children aged 8–15 years with applicability to a broad range of oral conditions [6]. Although the COHIP has been used to measure significant change in OHRQoL for children receiving surgery for cleft lip/palate, statistically significant change may not adequately assess whether a clinical intervention has a qualitative impact on the patient. The minimally important difference (MID), defined as “the smallest difference in score in the domain of interest which participants perceive as beneficial” [5, 7], can be used as a complementary, subjective tool for clinical assessment of meaningful improvement in patients [8–11].

Previously, children with orofacial anomalies reporting large clinical change were shown to have higher scores for individual COHIP domains (e.g., oral health, functional and emotional well-being) [5, 12]. However, the COHIP MID has not been reported. Further, MID methods in dental research are generally underutilized [8]. Thus, the primary objective of this study was to estimate the minimally important difference for the COHIP in children with craniofacial conditions using both the anchor and distributional methods.

## Methods

Data for analysis were derived from a 6-year, multi-center, prospective longitudinal study of youth with cleft conducted from 2009 to 2015. Youths and their caregivers participating in this study were followed at one of six major cleft treatment centers from the United States, including New York University Langone Medical Center, Children’s Hospital of Philadelphia, Lancaster Cleft Palate Center, Children’s Healthcare of Atlanta, University of Illinois-Chicago, and University of North Carolina-Chapel Hill. Participants included any child having a cleft lip and palate or cleft palate only between 7.5 and 18.5 years of age who spoke English or Spanish. Children who had a diagnosis of either an incomplete cleft lip without cleft of the alveolus, craniofacial syndrome or other complex medical conditions were excluded from the study. Participants were assessed at baseline and observed over two or three subsequent follow-up visits. The average length of time observed in the study for participants was 414 days, and the length of time between follow-ups ranged from 6 months to two years. During the course of the study, some patients received a surgical intervention and some did not. The primary objective of the parent study was to evaluate the effects of surgery for cleft lip/palate on psychosocial functioning, including depression, anxiety and resiliency. The secondary objective was to assess change in oral health related quality of life using the COHIP. Details of the study design, including study sample and surgical procedure descriptions, are available in a separate publication [13]. Analyses from this study do not evaluate the effects of surgical interventions for cleft lip/palate.

### Inclusion criteria

Participants who were present at baseline and the first follow-up observation, were between the ages of 7.5 and 18 years, and had not received a surgical intervention in the intervening time between visits were included in analysis. Participants were required to have complete COHIP data at baseline and first follow-up and complete data for the Global Assessment of Change at first follow-up. Eligibility criteria resulted in a final analytic sample of *N* = 281.

### Measures

#### COHIP

The Child Oral Health Impact Profile is a 34-item questionnaire designed to measure self-reported OHRQoL in children aged 8–15 years. The COHIP includes five domains, consisting of oral health (ten items), functional well-being (six items), social-emotional well-being (eight items), school environment (four items) and self-image (six items). There is also a final global health perception item. The COHIP has been previously shown to have good scale reliability, test-retest reliability and discriminant validity [5]. Response options for COHIP items include ‘never’ = 1, ‘almost never’ = 2, ‘sometimes’ = 3, ‘fairly often’ = 4, and ‘almost all the time’ = 5. Thus, overall COHIP scores could range from 34 to 170. Global health perception was assessed using a 5-point scale including ‘Poor’, ‘Fair’, ‘Average’, ‘Good’ and ‘Excellent’.

Subjects participating in the cleft study were asked to complete the COHIP at each scheduled observational visit. The COHIP was self-administered. Research Assistants were available to facilitate administration if participants needed additional help, though this was rare. The COHIP was offered in both English and Spanish. Following established procedures, the questions for oral health, functional well-being, social-emotional well-being and school environment were reverse-scored. Questions in each domain were summed, with higher scores indicating more positive OHRQoL. Overall COHIP scores were computed as a simple sum of all domain scores.

#### Global assessment of change

At each follow-up visit, study participants completed a Global Assessment of Change (GAC) questionnaire, which was used as the anchor in calculating the minimally important difference (MID) [8]. For each COHIP domain, participants were asked if they perceived any overall change in perception or functioning from the previous visit. For example, GAC for total health was assessed using the item “In general, has there been a change in your overall health since your last visit?” Participants then ranked their perceived change from the previous visit according to a 15-point global health transition scale, ranging from ‘A very great deal worse’ to ‘A very great deal better’ (Table 1) [14]. The GAC items used for each COHIP domain are summarized in Table 2.

**Table 1:** Global health transition scale used for Global Assessment of Change

7 A very great deal better	
6 A great deal better	
5 A good deal better	
4 Moderately better	
3 Somewhat better	
2 A little better	
1 About the same, hardly any better at all	
0 No change	
−1 About the same, hardly any worse at all	
−2 A little worse	
−3 Somewhat worse	
−4 Moderately worse	
−5 A good deal worse	
−6 A great deal worse	
−7 A very great deal worse

**Table 2:** Global Change Assessment questions for COHIP domains

COHIP Domain	GAC Item
Oral Health	Overall, has there been a change in the health of your teeth or mouth since your last interview?
Functional Well-being	Overall, has there been a change in the things your teeth or mouth do-like talking and/or eating-since your last interview?
Social Emotional Well-being	Overall, has there been a change in how you feel around your friends and family because of your teeth, face, or mouth since your last interview?
School Environment	Overall, has there been a change at school because of your teeth, face, or mouth since your last interview?
Self-Image	Overall, has there been a change in how you feel about yourself because of your teeth, mouth, or face since your last interview?
Total health	In general, has there been a change in your overall health since your last visit?

### Data analysis

Descriptive statistics were obtained for the analytic sample for select socio-demographic variables, including gender, race/ethnicity, age, cleft lip/palate abnormality status, surgical group recommendation (e.g., recommended for surgeries for functional defects, functional and visible defects together, or a surgery recommendation not accepted by the patient), and insurance pay type. The analytic sample was compared to children that were present at baseline but did not return for their follow-up visit on select demographic variables. Prior surgery histories for each participant were estimated based on a review of the medical records and parent reports. COHIP scores for each domain were obtained for the sample, as well as for the overall COHIP score (means, standard deviations and minimums/maximums).

The minimally important difference was calculated using the anchor and distribution criterion methods [8]. The Global Assessment of Change was used as the anchor. For each COHIP domain scale, global change was categorized as: ‘No Change’, defined as a GAC score of 0, −1 and 1; ‘Minimal Change’, defined as a GAC score of an absolute value of 2–3; and ‘Large Change’ defined as a GAC score of an absolute value of 4–7. Thus, if a patient indicated that they felt either “No change” or “About the same, hardly any better/worse at all” since the previous visit, they were assigned a global change score of “No Change”. For each GAC category (No Change, Minimal Change, etc.), the per-participant average change from baseline to 1st follow-up for each COHIP domain was calculated. The difference in COHIP change scores from the ‘Minimal Change’ and ‘No Change’ GAC categories was used as the Minimally Important Difference (clinically meaningful change). Following MID estimation for overall global change, GAC categories were stratified into positive and negative change and corresponding MIDs were re-calculated.

For the distribution criterion approach, we used the standardized effect size (ES) statistic, endorsed by the Cochrane Collaboration [8]. The ES statistic is calculated as the mean change in the COHIP from baseline divided by the standard deviation of the baseline estimate: *ES* = [(*m*_2_ − *m*_1_)/*s*_1_]. As previously described, a standardized ES of 0.2–0.5 is considered small, 0.5–0.8 as moderate and >0.8 as large [8]. ES statistics were calculated for each COHIP domain. The standardized response mean (SRM), defined as the mean difference of the change score divided by its standard deviation, was also calculated for each COHIP domain.

## Results

The analytic sample was approximately 57 % male and 53 % aged 12 years or older (Table 3). The sample was predominantly white (62.2 %) and had cleft lip and palate (80.8 %) as compared to cleft palate only (19.2 %). The average prior surgery history was 4.5 surgeries prior to the start of the study (baseline), with a standard deviation of 2.6. Compared to study participants who only presented at baseline, the analytic sample was not significantly different across gender or age, but was significantly different with respect to race/ethnicity and whether participants had cleft lip and palate or cleft palate only (data not shown). The average COHIP oral health domain score (Table 4) was 35.6 (SD = 6.5), followed by functional well-being (24.4, SD = 4.4), social-emotional well-being (31.6, SD = 7.4), school-environment (17.4, SD = 2.9), and self-image (22.7, SD = 4.6). Overall COHIP scores had a mean of 131.7 with a standard deviation of 18.9.

**Table 3:** Descriptive statistics of the analytic sample at baseline

Variables	*N*	%
Gender		
Female	120	42.7
Male	161	57.3
Race/Ethnicity		
White	173	62.23
Hispanic	47	16.91
Black	21	7.55
Asian	30	10.79
Other	7	2.52
Age		
< 12 years	132	46.98
12+ years	149	53.02
Cleft Lip/Palate		
CLP	227	80.78
CPO	54	19.22
Surgical Group		
Visible + Both	19	7.06
Invisible	21	7.81
Not accepted	229	85.13
Paytype		
Private	167	62.08
Non-Private	102	37.92
	Mean	SD
Prior Surgery History	4.53	2.62

**Table 4:** Summary statistics of the Child Oral Health Impact Profile at baseline (*N* = 281)

COHIP Scale	*N*	Mean	SD	Min	Max
Oral Health	281	35.64	6.51	16	50
Functional Well-being	281	24.35	4.43	11	30
Social Emotional Well-being	281	31.61	7.35	8	40
School Environment	281	17.39	2.86	4	20
Self-Image	281	22.68	4.64	6	30
COHIP Overall	281	131.68	18.87	47	167

MID estimate results for the anchor-based approach using the Global Assessment of Change scale (Table 5) indicate that the minimally important difference ranged from −0.32 to 2.95. The mean change from baseline for each COHIP domain stratified by GAC change categories is also shown. MID estimates were 0.15 for oral health, −0.32 for functional well-being, 0.12 for social-emotional well-being, 0.84 for school environment and 0.60 for self-image. The MID for the overall COHIP was 2.95. Effect size statistic estimates for these COHIP domains were 0.16, 0.12, 0.22, 0.21 and 0.19, respectively. Effect size estimates for the overall COHIP was 0.25. Results for standardized response means of each domain were similar to effect sizes.

**Table 5:** Minimally Important Difference (MID) of the Child Oral Health Impact Profile, 4-point anchor method, effect size (ES) statistic and standardized mean response (SRM)

	Global Assessment of Change							Effect Size		SRM
	−1,0,1		2–3^a^		4–7		MID			
COHIP Domain	*N*	Mean Change	*N*	Mean Change	*N*	Mean Change	Change	*N*	Estimate	Estimate
Oral Health	114	0.93	71	1.08	96	1.15	0.15	281	0.16	0.16
Functional	176	0.41	58	0.09	47	1.53	−0.32	281	0.12	0.13
Social Emotional	191	1.29	32	1.41	58	2.84	0.12	281	0.22	0.27
School Environment	204	0.38	36	1.22	41	1.10	0.84	281	0.21	0.25
Self-Image	177	0.93	49	1.53	55	0.07	0.60	281	0.19	0.22
COHIP Overall	175	4.17	41	7.12	65	4.43	2.95	281	0.25	0.31

^a^Compared to no change in GAC to calculate the MID

Stratified Global Assessment of Change scores (Table 6) indicate that overall anchor-based MIDs for the COHIP score were 3.29 and −10.17 for positive and negative change, respectively. Negative MIDs for individual COHIP domains included −0.93 (oral health), −1.86 (functional well-being), −0.29 (social-emotional well-being), −0.18 (school environment), and 1.07 (self-image). For positive MIDs, scores included 0.24 (oral health), 0.06 (functional well-being), 0.19 (social-emotional well-being), 1.01 (school environment), and 0.54 (self-image).

**Table 6:** Minimally Important Difference (MID) of the Child Oral Health Impact Profile vs better or worse global change

	Global Assessment of Change											
	Worse				No Change		Better					
	(−7)–(−4)		(−3)–(−2)		−1, 0, +1		2–3		4–7		MID-	MID+
COHIP Domain	N	Mean Change	N	Mean Change	N	Mean Change	N	Mean Change	N	Mean Change	Change	Change
Oral Health	3	0.67	5	0.0	114	0.93	66	1.17	93	1.16	−0.93	0.24
Functional	1	0.0	11	−1.45	176	0.41	47	0.47	46	1.57	−1.86	0.06
Social Emotional	1	9.00	5	1.00	191	1.29	27	1.48	57	2.74	−0.29	0.19
School Environment	4	0.50	5	0.20	204	0.38	31	1.39	37	1.16	−0.18	1.01
Self-Image	4	−6.50	6	2.00	177	0.93	43	1.47	51	0.59	1.07	0.54
Overall	2	−3.00	1	−6.00	175	4.17	40	7.45	63	4.67	−10.17	3.28

## Discussion

Most quantitative research, including quality of life research, use tests of statistical significance to interpret findings and study results. However, statistical significance in OHRQoL measures does not identify whether changes achieved have a qualitative impact on the patient [15], and large sample sizes can reveal statistically significant differences that may not be clinically meaningful or relevant to the patient [16]. Since a statistically significant change might not indicate real effects on patients’ lives, there has been a growing trend in quality of life outcomes research to measure clinically meaningful change using minimally important differences [8]. Instead of defining change on the basis of a statistical test of mean scores, MID uses the subjective perspective of the patient to determine what kind and how much change is meaningful [10, 17]. Despite MID’s utility as a clinically meaningful and sensitive assessment of change over time, it is rarely utilized in oral health and cleft research [18].

This study is the first to identify the MID of the Child Oral Health Impact Profile, a validated measure of oral health-related quality of life, in youth with orofacial anomalies. The sample included youth followed for ongoing assessments at cleft treatment centers and purposefully included only those seen for annual evaluations and not receiving a surgical intervention during the initial study period. COHIP MIDs provide a valuable tool for interpreting clinically meaningful change in OHRQoL in youth with cleft over time. However, it is also important to compare minimally important differences with other samples of youth with cleft, as well as youth with other oral conditions or who receive alternate treatment. Further, it is recommended that MID estimates are compared with qualitative data to obtain a more comprehensive understanding of patients’ subjective experiences with cleft treatment over time [19]. Finally, comparing youth perceptions of quality of life change with proxy ratings by caregivers can identify the level of agreement with external subjective evaluations [20].

The study findings may have important clinical and treatment implications. MID estimates can be used pre and post cleft-related surgery to determine the impact of particular surgery types, as well as the optimal timing of surgical interventions on youth OHRQoL. While all cleft centers follow Parameters of Care established by the American Cleft Palate-Craniofacial Association ^21^, there is wide variation across individual centers regarding the amount and timing of surgery recommended and completed with patients ^22^. Further, preliminary unpublished results from the parent study of children with cleft indicate that there may be diminishing returns for those patients undergoing more surgery than others with the same condition. Therefore, determining the type and timing of surgical interventions (e.g., orthognathic versus lip/nose revisions; childhood versus adolescence) that culminate in the most positive clinically meaningful change for patients could have substantial ramifications for cleft care.

Despite their usefulness, there are some disadvantages to using anchor-based methods to determine the MID. Anchor-based methods fail to consider instrument precision, their reliability is unknown, and they are influenced by a specific rating scale and anchors ^23^. Measurement error due to recall bias and confounding by response shift are additional concerns. Finally, the validity and reliability of global change measures is suspect, as is valid self-judgment of change over time ^8,24^. For these reasons and following established recommendations, we provided estimates of the Minimally Important Difference through both anchor-based and distribution methods, including the effect size statistic and standardized response means. However, there are alternative distribution-based measures of MIDs, including the standard error of measurement (SEM), paired t-statistic, and half standard deviation. Thus, different distribution methods of MIDs, as well as the choice of anchor used for estimates of global assessments of change, may yield varying results [8]. While the SEM measure incorporates instrument reliability in its calculation, and is therefore not sample dependent, it does not have a simple interpretation like that of standard effect sizes [11]. Due to its popularity and robustness to homogeneity and heterogeneity in sample data [8, 11], the effect size statistic is appropriate for this patient population.

Further limitations stem from the observational design of the parent study. There were no specific inclusion criteria regarding where participants were in the treatment process, the number of prior surgeries received, or appearance, speech proxy, or professional ratings across sites. Further, while the majority of youth with cleft in the US are followed by registered teams with the American Cleft Palate-Craniofacial Association that have experienced surgeons, team philosophies regarding treatment activism is not controlled. Finally, as the analytic dataset used in this study compared COHIP change from baseline to 1st follow-up, any participants who were present at baseline but did not return for follow-up evaluations were not included in analysis. For this study, children who were lost to follow-up after their baseline observation were significantly different from the analytic sample with respect to race and whether children had cleft lip and palate or cleft palate only. Thus, the generalizability of MID results may be further limited.

In conclusion, this research provides an important contribution to the study of OHRQoL and cleft care by identifying MIDs for the overall COHIP and its domains. These estimates can be used to assess clinically meaningful change in OHRQoL among youth with cleft over time. Future research can benefit from comparing MIDs between children with cleft and/or palate who received surgery and continued throughout post-operative follow-ups to those who did not receive a surgery recommendation. Additionally, continued follow-up with participants for which no surgery is recommended or rendered into adulthood may provide additional insight into meaningful change over time.
